# Supplementary material for: Changes in key recruitment performance metrics from 2008–2019 in industry-sponsored phase III clinical trials registered at ClinicalTrials.gov
Source: PLoS One. 2022 Jul 26;17(7):e0271819. doi: 10.1371/journal.pone.0271819 (PMC9321424; doi:10.1371/journal.pone.0271819)
Supplement: Attachment — Submitted filename: response letter.docx [file pone.0271819.s001.docx]

Dept. of Dermatology

Bispebjerg Hospital

DK-2400 København NV

Zarqa_ali@hotmail.com

PLOS ONE

June 28^th^ 2022

Re: PONE-D-22-12003 “Changes in Key Recruitment Performance Metrics from 2008-2019 in Industry-sponsored Phase III Clinical Trials Registered at ClinicalTrials.gov” by Brøgger-Mikkelsen et al.

**Dear Editor-in-Chief**

Thank you for giving us the opportunity to revise our paper and for the valuable suggestions from the reviewers. We have revised the paper according to the suggestions and issues raised.

**Journal requirements:**

**Comment 1:**

Please ensure that your manuscript meets PLOS ONE's style requirements, including those for file naming.

**Author response:**

The manuscript has been revised carefully to meet PLOS ONE’s style requirements. We hope the manuscript is suitable for PLOS ONE now.

**Comment 2:**

Thank you for stating the following financial disclosure:

"No funders had any role in study design, data collection and analysis, decision to publish, or preparation of the manuscript."

At this time, please address the following queries:

a) Please clarify the sources of funding (financial or material support) for your study. List the grants or organizations that supported your study, including funding received from your institution.

b) State what role the funders took in the study. If the funders had no role in your study, please state: “The funders had no role in study design, data collection and analysis, decision to publish, or preparation of the manuscript.”

c) If any authors received a salary from any of your funders, please state which authors and which funders.

d) If you did not receive any funding for this study, please state: “The authors received no specific funding for this work.”

Please include your amended statements within your cover letter; we will change the online submission form on your behalf.

**Author response:**

The authors received no specific funding for this work. This statement has been added to the cover letter and thank you for changing the online submission on our behalf.

**Comment 3:**

We note that you have indicated that data from this study are available upon request. PLOS only allows data to be available upon request if there are legal or ethical restrictions on sharing data publicly. For more information on unacceptable data access restrictions, please see http://journals.plos.org/plosone/s/data-availability#loc-unacceptable-data-access-restrictions.

We will update your Data Availability statement on your behalf to reflect the information you provide.

**Author response:**

The link to all raw data is https://aact.ctti-clinicaltrials.org/connect. All the data is publicly available and with use of the provided link it is possible to get access to data used for analyses in the present manuscript.

**Comment 4:**

Please upload a new copy of Figures 3, 4 and 6 as the detail is not clear. Please follow the link for more information: https://blogs.plos.org/plos/2019/06/looking-good-tips-for-creating-your-plos-figures-graphics/"" https://blogs.plos.org/plos/2019/06/looking-good-tips-for-creating-your-plos-figures-graphics/

**Author response:**

A new copy of figure 3, 4, and 6 has been provided in the required format.

**Comment 5:**

Please review your reference list to ensure that it is complete and correct. If you have cited papers that have been retracted, please include the rationale for doing so in the manuscript text, or remove these references and replace them with relevant current references. Any changes to the reference list should be mentioned in the rebuttal letter that accompanies your revised manuscript. If you need to cite a retracted article, indicate the article’s retracted status in the References list and also include a citation and full reference for the retraction notice.

**Author response:**

The reference list has been reviewed to ensure it is complete and correct.

**Reviewers' comments:**

**Reviewer #1:**

**Comment 1:**

Interesting paper but I could do with less statistical analysis and significance testing.

The generic question of interest is whether the drug industry is doing less trials over time. That is true from your results for multicenter trials but is it true overall for all industry sponsored trials?

**Author response:**

Thanks for rising this interesting comment. Our results suggest that there is a reduction in multicenter trials; however, this is not true for all industry sponsored trials. It would be interesting to examine that more in the future. We have elaborated this issue in the section of limitation.

**Comment 2:**

My horseback counts from ClinicalTrials.gov for industry sponsored trials completed 2008-2019 is 64,537 and 15,517 for multicenter trials. Counts corresponding to your Table 1 for all industry sponsored trials are 20,092, 21,297, and 23,146 and for multicenter trials were 4,633, 5,090, and 5,794. You would do well with an introduction as to how you get from these numbers to those focused upon in your paper and also if these results reinforce your conclusions.

**Author response:**

Thanks for this comment. We have not included all industry-sponsored trials but only phase III trials with a drug as an intervention. We have emphasized that in the last section of introduction, first section of methods, and first section of discussion.

**Reviewer #2:**

**Comment 1:**

Add related works as a separate section

**Author response:**

Related work has been added as a separate section in the discussion.

**Comment 2:**

Contributions of the research can be added at the end of introduction section

**Author response:**

Author contribution has been added at the end of introduction.

**Comment 3:**

Conclusion should be added at the end of the manuscript providing overall summary of the manuscript

**Author response:**

Thank you for this comment. Conclusion section has been added at the end of the manuscript and revised to provide an overall summary of the manuscript.

**Comment 4:**

Provide clear images for all the Figures

**Author response:**

Thanks for making us ware of it. Clear images for all the figures have been provided.

**Comment 5:**

Line 188, As seen in figure 2b, trial duration increased significantly pairwise between all three time intervals. Significantly pairwise?

If possible add Exploratory Data Analysis

**Author response:**

Thank you for this comment. We acknowledge that the sentence may be confusing. The sentence has been rephrased and “pairwise” has been deleted.

Further, we recognize the merit of exploratory data analysis, but for the purpose of this study, we believe that our aggregate data analysis in the tables and figures represents data well.

**Comment 6:**

Which users benefit from this study?

**Author response:**

Thanks for this interesting question. Researchers and academics trying to understand recruitment patterns would benefit from this work. Private recruitment companies will also benefit from better understanding of changes in the recruitment over time.

Looking forward to hearing from you,

Yours sincerely

Zarqa Ali, MD PhD

Dept. of Dermatology

Bispebjerg Hospital

DK-2400 København NV

Zarqa_ali@hotmail.com
